# Structural equation model of the relationship between functional ability, mental health, and quality of life in older adults living alone

**DOI:** 10.1371/journal.pone.0269003

**Published:** 2022-08-03

**Authors:** YuMi Yi, Yeon-Hwan Park

**Affiliations:** 1 Department of Nursing, College of Natural Science, Dong-Eui University, Busan, Republic of Korea; 2 College of Nursing, The Research Institute of Nursing Science, Seoul National University, Seoul, Republic of Korea; 3 College of Nursing, Seoul National University, Seoul, Republic of Korea; Universita degli Studi di Firenze, ITALY

## Abstract

**Aims:**

Living alone, a reality in an increasing number of older adults recently, is a risk factor for low quality of life. This study identified the predictors of quality of life in older adults living alone based on mental health and the International Classification of Functioning, Disability, and Health.

**Methods:**

This secondary data analysis used information from the 2018 Assessing the Requirements of Customized Health Care and Daily Living Support Services survey (N = 1,022), collected from adults aged ≥ 65 living alone in Siheung City, South Korea, from August to October 2018. The exogenous variables were body functions (hand grip strength, timed “up and go” test score, and body mass index), daily living activities (Korean Instrumental Activities of the Daily Living Scale), social activity participation (social activity engagement, neighbor contacts, and family contacts), and participation in economic activity (frequency). The endogenous variables were mental health (Geriatric Depression Scale Short Form—Korean Version and UCLA Loneliness Scale) and quality of life (EuroQoL-5 Dimension-3 Level and EuroQoL-Visual Analog Scale).

**Results:**

After modifying the hypothetical model, which had failed to satisfy the recommended fitness level, the (modified) model had good fitness indices Q (CMIN / df) 2.90, GFI 1, AGFI 1, RMSEA 0.04, CFI 0.90 and PCFI 0.53. Of the nine pathways of the modified model, five were statistically significant. Quality of life was affected by body functions, daily living activities, social activity participation, and mental health. These variables explained 68.2% of the factors affecting quality of life.

**Conclusions:**

By highlighting the role of mental health, this model provides a useful framework for improving the quality of life of older adults who live alone and function at various levels in the community. Focusing on advancing mental health through body functions, daily living activities, and social activity participation can improve quality of life.

## Introduction

The number of older adults living alone has been increasing rapidly in recent years owing to accelerated lifestyle changes, extended life expectancy, and changes in family relationships and living conditions. The ratio of older adults living alone rose from 11% to 15% for women and from 6% to 8% for men between 1990 and 2010 [1]. In South Korea, this change was more pronounced, climbing from 3.8% in 2000 to 7% in 2017 [2]. As the population ages, the long-term goal of aging populations is to pursue value in life [3]. Quality of life (QoL) is an individual’s perception of his/her position in life in relation to his/her culture and value system, goals, expectations, standards, and concerns [4]. It is a multi-dimensional variable that encompasses multiple domains, including health and function, mental well-being, social relations and activities, home and neighborhood, independence and freedom, and financial conditions [5]. Therefore, maintaining and promoting QoL is a key goal of health intervention for older adults [6,7].

Older adults often face health problems associated with aging, financial difficulties due to reduced income and economic dependence, loneliness caused by social and psychological conflicts and breakdown of relationships, and lethargy due to the loss of social roles [8]. Living arrangements can have a profound effect on older adults’ QoL and mental health [9,10]. Living with others means having readily available social and personal resources and is an influential factor in the pattern of everyday social interactions [11]. Living alone can cause a lack of social cohesion due to changes in family relationships [12], social isolation, and reduced social support [13]. Thus, older adults living alone are more vulnerable and need special attention compared to older adults living together because they are at increased risk of physical and mental health deterioration [8,14–16].

Most older adults value living independently in a familiar environment where they feel they belong [17,18]. Older adults living alone must have the appropriate functional abilities to manage basic everyday problems and access community care and medical services in order to pursue a high QoL independently in their community [19–22].

The International Classification of Functioning, Disability and Health (ICF) is a model that focused on three main categories of functional abilities: body functions and structure, activity, and participation. The ICF model recognizes older adults as beings that require participation in daily activities throughout their lives [23]; therefore, when studying the QoL of older adults living alone, it is useful to consider the functioning ability they require to live independently [24]. Prior studies using the ICF model [25–27] have explained QoL with a focus on functional ability but have not sufficiently accounted for the emotional components. Emotions are the main indicators of subjective well-being [28], and for older adults, dissatisfaction or discomfort with the past is more dominant than hope for the future [29]. In particular, older adults living alone tend to have a notable decline in the mental domain of their QoL [30]. Living alone is independently involved in reduced QoL by increasing the risk of depression and loneliness [31]. Thus, depression and loneliness should be included as factors affecting QoL.

Studies on QoL for older adults living alone have mainly focused on research that identified influential factors [32,33] and the effects of services or programs [34,35]. These studies have only analyzed a fragmentary understanding of factors affecting QoL or a correlation between related factors and QoL [36,37]. As there are increasing numbers of older adults living alone who have complex chronic diseases but are in good health [38], it is important to identify various factors that affect the QoL of those with varying levels of functionality in communities. For this purpose, structural equation models were used in this study because they allow for the simultaneous analysis of the interrelationships of independent variables and their indirect effects through other variables.

The QoL of older adults living alone is a very important concept not only for individual but also for public health and health policy decisions. In particular, older adults living alone can maintain an independent and valuable life for as long as possible within the societies to which they belong when they possess the functional abilities and mental health needed to take care of themselves. Therefore, it is very necessary to understand the functional ability and mental health of older adults living alone. Based on this necessity, this study aimed to comprehensively identify the relationships between factors affecting the QoL of older adults living alone and demonstrate the direct and indirect relationship between these factors by establishing a hypothetical model including mental health-related concepts based on the functional ability aspect of the ICF model. The findings were expected to provide guidance for assessing and predicting the QoL of older adults living alone when implementing community care practices and to provide the basic data needed for the development of interventions to improve their QoL.

## Materials and methods

### Design

We implemented a cross-sectional design through secondary data analysis to construct and verify a hypothetical model of the QoL of older adults living alone.

### Participants and ethical considerations

Participants were 1,022 older adults living alone who participated in the first year of the Assessing the Requirements of Customized Health Care and Daily Living Support Services survey [39], conducted with the support of the Korea Health Industry Development Institute in Siheung City, Gyeonggi-do, from 2018 to 2020.

The final sample included 27% of the total population of 3,753 older adults living alone in Siheung city. Inclusion criteria were (a) being aged ≥ 65 years, (b) living alone in Siheung city, and (c) being able to communicate orally and provide written informed consent. A total of 1,041 participants were selected through convenience sampling for each of the 16 regions in Siheung city; 19 participants were excluded owing to not living alone and four questionnaires had incomplete answers. Participants were recruited to take part in interviews and a physical measurement session. A total of 50 people volunteered as research assistants to administer the questionnaire and take physical measurements of the participants. Prior to data collection, research assistants were trained in research (purpose, contents, evaluation method) and research ethics. The evaluation method was clarified through a detailed document explaining the research tools and a question–answer session after a two-hour training session. To secure reliability between the measurers, the researcher and research assistants measured two identical subjects at the same time and made adjustments until there were no discrepancies between the assessors. The respondents completed the survey within approximately 50 min.

Approval was obtained before conducting each of the two studies from the respective institutional review boards (IRB). The original data were collected with the approval of Seoul Hospital IRB (IRB NO. H-1807-131-961). The study involving secondary analysis was conducted after obtaining approval (IRB No. E1902/002-001) from the Research Ethics Committee of Seoul University. The original study participants signed informed consent prior to the study; they were informed that their participation was voluntary and that they had the right to withdraw from the study without penalty or loss of any privileges. The original informed consent included information that the data collected in the study could be used in other studies. Therefore, written informed consent was not required in the secondary analysis; informed consent was implied for participants in the original study. However, this study excluded one participant who did not provide written consent to use the secondary data.

### Hypothesized framework

**Fig 1:**
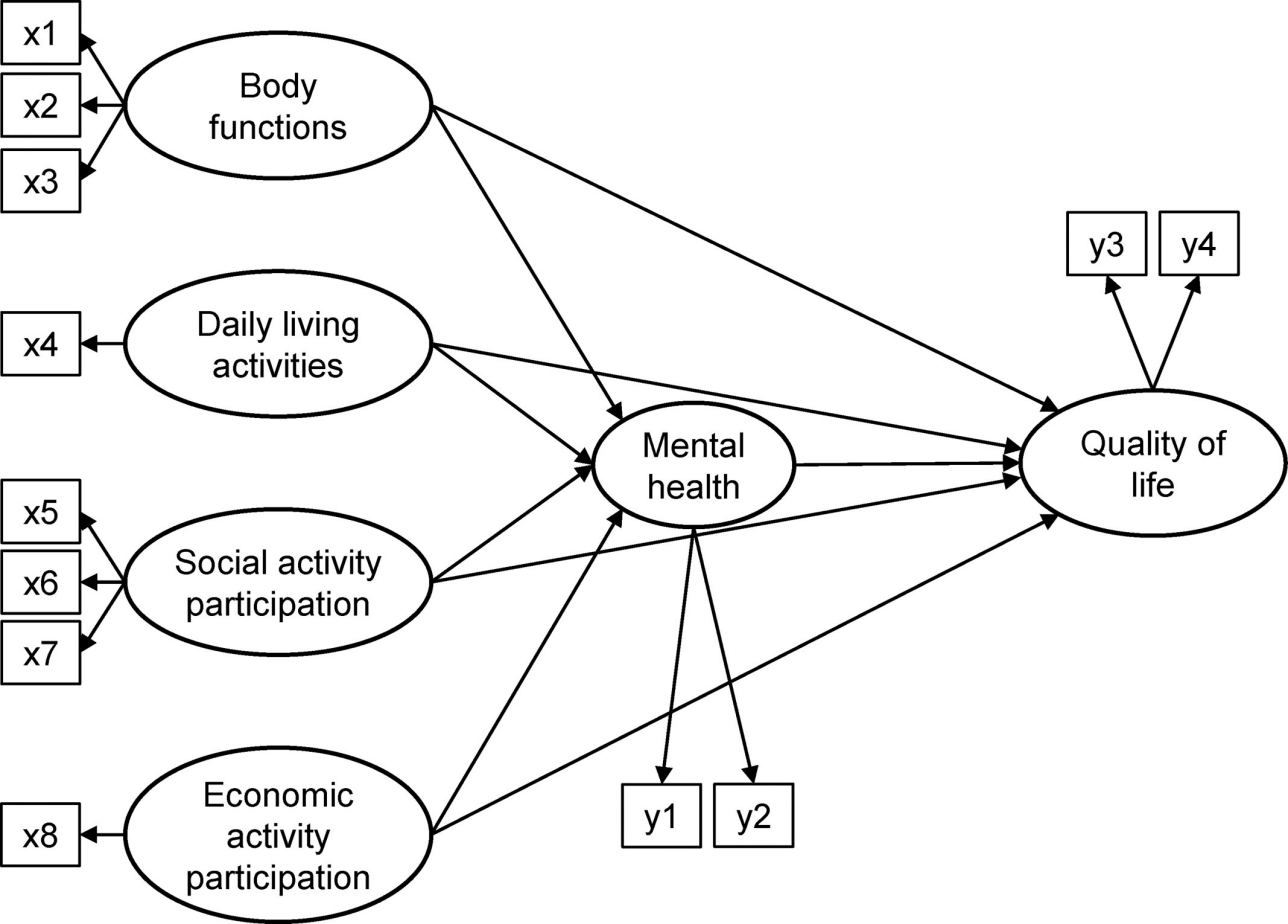
Hypothetical model. x1 Hand grip strength; x2 Timed up and go score; x3 Body mass index; x4 Korean instrumental activities of daily living; x5 Social activity participation frequency; x6 Number of neighbor contacts; x7 Number of family contacts; x8 Economic activity participation frequency; y1 Geriatric Depression Scale Short Form-Korean; y2 Revised UCLA Loneliness Scale-version 3; y3 Euro Quality of Life-5 Dimension index; y4 Euro Quality of Life Visual Analogue Scale.

For the hypothesized framework, we used key concepts from the WHO’s ICF model and mental health factors related to the QoL of older adults living alone identified in extant literature reviews. The ICF model explains the factors constituting health conditions by dividing them into two categories: functioning and disability, and contextual factors. Functioning and disability interact with contextual factors. The ICF model includes three components of functioning and disability: body functions and structure, activity, and participation. The contextual factors consist of personal and environmental factors [23]. Since the ICF model describes health through functional abilities, it can be assumed that the goal of a healthy life, QoL, will improve as activity and participation increase. We sought to adapt a reduced version centered on the functional capabilities of the ICF model to identify QoL according to various functional levels of older adults in our study: body functions and structure, activity, and participation. This is also consistent with the fact that to increase the value and explanatory power of a model, relationships between as few causal path concepts as possible should be established [40]. For the older adults living alone to remain independent in their communities, it is important to maintain muscle mass and strength [41] and sufficient mobility to move safely [42]. Hand grip strength (HGS) is a simple and efficient method to assess overall muscle strength, nutritional status, and muscle mass [43,44]. The timed up and go (TUG) test is reliable, safe, and readily available for mobility and disability assessment [45]. The body mass index (BMI) is a useful indicator for screening the risk of functional limitation by indirectly measuring the amount of muscle mass or body fat [46,47]. We measured HGS, TUG, and BMI to assess body functions. Body structure information was not included in the raw data used in the current study. Activity was set using the instrumental activities of daily living (IADL), which is a multidimensional functional evaluation that requires a complex range of activities and contact with the outside, along with other measurements of daily living activities [48,49]. IADL has a greater impact on the independence of older adults than activities of daily living (ADL), as it evaluates how well individuals can adapt to their environment [50]. Participation included social activity participation (SAP) and economic activity participation (EAP). A higher level of activity participation reports enhanced QoL [51]. SAP is maintaining close relationships with others and continuing meaningful activities [52]. As SAP increases, physical functions become smoother [53] and social interactions become more frequent [54]. Therefore, SAP can serve as a buffer to the negative QoL of older adults living alone [55]. EAP means participation in paid work [56]. EAP has a significant impact on improving the QoL of older adults because it satisfies the basic need for livelihood, helps restore the sense of loss of role from job loss, and enhances the physical and mental satisfaction of older adults through social participation [57–59]. Mental health factors were verified to have a significant impact on the QoL of the older adults living alone through literature reviews [60]. Older adults living alone have increased incidences of loneliness due to difficulties in daily living and functional social isolation, which result from decreased functional ability. As a result, they experience a higher level of depression and lower QoL. Therefore, the primary focus of the hypothesized framework was on body functions, activity, participation, mental health, and QoL. Fig 1 shows how the ICF model and literature reviews were used to select variables and create a structural equation model, which was then used to interpret the study’s findings.

### Variables and measurements

#### Quality of life (QoL)

QoL was measured with the Euro QoL-5 Dimension-3 Level (EQ-5D-3L) and the EQ Visual Analogue Scale (EQ-VAS). Developed by EuroQoL group [61], the EQ-5D-3L is composed of five dimensions: mobility (M), self-care (SC), usual activities (UA), pain/discomfort (PD), and anxiety/depression (AD). Each dimension has three levels of severity, indicating no problems, some or moderate problems, and extreme problems. Levels of severity are labelled as 1, 2, 3 and the labels are used to form part of a numerical description of QoL. If the answer to all five dimensions of EQ-5D is 3, N3 = 1, otherwise, N3 = 0. The EQ-5D index is a numerical value converted to 1-(0.050 + 0.096 x M2 + 0.418 x M3 + 0.046 x SC2 + 0.136 x SC3 + 0.051 x UA2 + 0.208 x UA3 + 0.037 x PD2 + 0.151 x PD3 + 0.043 x AD2 + 0.158 x AD3 + 0.050 × N3); the higher the EQ index, the higher the QoL. At the time of development, Cronbach’s α was .88 for tool reliability. In this study, Cronbach’s α was .75.

The EQ-VAS tool is in the form of a 20 cm vertical line with 100 points at the top, representing the highest health state that can be imagined, and 0 at the bottom, indicating the lowest health state, and a score between 0 points and 100 points is given for the health state. The higher the EQ-VAS score, the better the subjective health status.

#### Depression (Geriatric Depression Scale Short Form Korean Version, GDSSF-K)

The Korean version of the Short Form Geriatric Depression Scale (GDS-15), modified by Kee [62], was a GDS abbreviated form, developed by Sheikh and Yesavage [63]. The GDSSF-K has 15 items answered using a yes/no response. Twenty items represent a depressed response with a “yes” answer, and 10 items indicate a depressed response with a “no” answer. Values on the scale range from 0 to 15, with higher values indicating more symptoms of depression. Participants with a total score of 5 or less are considered normal. At the time of development, the instrument’s Cronbach’s α was .88. In this study, Cronbach’s α was .87, and the regression coefficient of three items (2, 4, 14) was removed through confirmatory factor analysis, and 12 items were used to construct the model, with a Cronbach’s α of .87. Using construct validity analysis, a single factor was extracted, and the total explanatory power was 45.8%.

#### Loneliness (Revised UCLA Loneliness Scale, RULS)

Loneliness was assessed using the RULS, developed by Russel, Peplau, and Cutrona [64] and translated by Kim [65], which comprises 20 items rated on a four-point Likert scale ranging from 1 (never) to 4 (often feel this way), for a possible score ranging from 20 to 80. Higher scores reflect greater loneliness. Cronbach’s α for reliability at the time of tool development was .93. In this study, Cronbach’s α was .90, and 18 items were used for model construction except for two items (4 and 9) with standard regression coefficient less than 0.5. The Cronbach’s α was .91. Using construct validity analysis, three factors were extracted, and the total explanatory power was 55.4%.

#### Hand grip strength (HGS)

HGS is a portable, relatively inexpensive, and reliable measure of muscular strength. Participants held a dynamometer (TANITA No. 6103, Tokyo, Japan) in the hand to be tested, with the arm at a right angle and the elbow by the side of the body. The base rested on the first metacarpal (heel of palm), while the handle rested on the middle of the four fingers. Participants were then instructed and verbally encouraged to squeeze the hand grip as hard as they could, with the forearm held parallel to the body in the standing position. HGS for each hand was measured twice individually and then the mean value of all four measurements indicated the index of grip strength [66]. Low HGS was defined as < 26 kg and < 18 kg for males and females, respectively, using the Asian Working Group for Sarcopenia [67]. In this study, we applied the standard of decrease in grip strength; 0 indicated “normal” and 1 indicated “reduced” grip strength.

#### Timed up and go (TUG) test

The TUG test evaluates functional mobility along with movement and balance ability. While sitting in a chair with armrests, the time taken to sit again after standing up from the chair and walking 3 m, after the experimenter’s start signal, is measured using a digital stopwatch. We used aids if necessary and did not provide physical assistance. A longer time period means reduced functional state. In this study, 13.5 seconds or more was classified as representing a high-risk of falling [68]; that is, a score of 0 indicated the “no-risk group,” and 1 indicated the “high-risk group.” Inter-tester reliability (r = 0.99) and intra-tester reliability (r = 0.98) were reported to be high [69].

#### Body mass index (BMI)

The BMI determines the ideal weight with respect to height (kg/m^2^). In this study, height was measured up to 0.1 cm using the ultrasonic height meter (IKI HT001 No. 600513169, China) after verifying the height-zero correction and measurement position (whether heel, buttocks, back, and back of the head were in contact with the wall) when standing against a flat wall. If the height could not be measured in a normal position (owing to wheelchair, scoliosis, etc.), the demi-span was measured with a tape measure with both arms open. The weight was measured up to 0.1 kg after zeroing the scale with the shoes removed and the clothes as light as possible. If weight measurement was difficult (owing to use of wheelchair, etc.) in the normal way, the upper arm circumference, calf circumference, and waist circumference (cm) were measured and calculated. Obesity judgements were classified as low weight (BMI < 18.5), standard weight (18.5 ≤ BMI < 23), overweight (23 ≤ BMI < 25), and obesity (BMI ≥ 25) according to the obesity recommendation standards set by the Asia-Pacific Standard [70]. In this study, a score of 0 indicated “standard weight,” and 1 indicated “unusual weight” including low weight, overweight, and obesity. When measuring height, the concurrent validity between the InBody height meter and ultrasonic height meter was noted at the 0.01 level with a Pearson’s correlation coefficient of .99.

#### Korean Instrumental Activities of Daily Living (K-IADL)

The K-IADL tool developed by Won et al. [65] is a four-point scale with a total of 10 questions on grooming, household chores, preparing meals, laundry, going out—close range, using transportation means, buying things, managing money, using phones, and taking medicine. We contacted the original author by e-mail for approval to use the tool. The tool score ranges from 10 to 37; the lower the score, the higher the degree of instrumental daily activity independence and physical health. As for reliability at the time of development, Cronbach’s α was .94. In this study, Cronbach’s α was .81. Through confirmatory factor analysis, three items (1, 8, 10) with standardized regression coefficient ≤0.5 were removed, and seven questions were used for model construction. Cronbach’s α was .81. Using construct validity analysis, two factors were extracted, and the total explanatory power was 57.7%.

#### Social activity participation (SAP)

The items adopted in the social activities section of the structured Health and Welfare Ministry’s Survey Card of Elderly Living Alone [71], rated on a four-point Likert scale, consisted of three points for “3 times or more per week,” two points for “1–2 times per week,” one point for “1–2 times per month,” and zero points for not participating. The answer to the question on the number of neighbor contacts and number of family contacts consisted of four points for “more than 1–2 times per week,” three points for “1–2 times per month,” two points for “1–2 times per quarter,” one point for “1–2 times per year,” and zero points for not participating. A higher score means a higher frequency of participation.

#### Economic activity participation (EAP)

The items adopted in the economic activities section of the structured Health and Welfare Ministry’s Survey Card of Elderly Living Alone [71] were rated on a four-point Likert scale. Three points were awarded for “3 times or more per week,” two points for “1–2 times per week,” one point for “1–2 times per month,” and zero points for not participating. A higher score means a higher level of participation.

### Data analysis

The data were analyzed using SPSS version 23 and AMOS version 23 software. SPSS was used for the participants’ descriptive statistics (characteristics) and research variables. Normality of the data was confirmed through univariate skew, kurtosis, and multivariate kurtosis. Correlation and multicollinearity between variables were analyzed with Pearson’s correlation coefficients and variance inflation factor (VIF). Confirmatory factor analysis was used to confirm the validity of latent variables. We used the asymptotically distribution-free (ADF) estimation to test the fit and hypothesis of the structural model. The following parameters were used to estimate model fit: χ^2^/df ≤3.00, GFI ≥ 0.90, AGFI ≥ 0.90, RMSEA ≤ 0.08, CFI ≥ 0.90, NFI ≥ 0.90, IFI ≥ 0.90, and a lower PNFI [62]. The bootstrap method was used to verify the statistical significance of the indirect and total effects of the model.

## Results

### Participant characteristics

The participants’ characteristics are shown in Table 1. The sample comprised 1,022 community-dwelling older adults living alone (77.9% female; 22.1% male). The average age was 76.2 years (range: 65–102 years). The majority (76.3%) were widowed, 938 (91.8%) had living children, and the average number of living children was 2.8. Of the respondents, 38.8% had no formal education, the average number of chronic diseases was over 5, and 664 (65.0%) followed a religion. The average household monthly income was about 554,333 Korean won, and the monthly living expenses equaled an average of 517,549 Korean won.

**Table 1:** General characteristics of the participants (N = 1022).

Category		n	(%)
Gender	Male	226	22.1
	Female	796	77.9
Age (years)	65–74	402	39.3
	75–84	547	53.5
	≥ 85	73	7.2
	Mean±SD	76.2±5.9	
Marital status	Single	28	2.7
	Married	10	1.0
	Divorced/separated	204	20.0
	Widowed	780	76.3
Living child	Yes	938	91.8
	No	84	8.2
	Mean±SD	2.8±1.6	
Level of education	Illiterate	396	38.8
	Elementary school	307	30.0
	Middle school	148	14.5
	High school	129	12.6
	≥ College	42	4.1
Religion	Yes	664	65.0
	No	358	35.0
Monthly income (Korean won)	Mean±SD	554,333±390,994	
Monthly living expenses (Korean won)	Mean±SD	517,549±393,682	
Number of chronic diseases	0	26	2.5
	1	66	6.5
	2	88	8.6
	≥ 3	842	82.4
	Mean±SD	5.22±3.0	

SD = standard deviation.

### Descriptive statistics, normality, and multicollinearity of the study variables

Table 2 shows the mean, standard deviation, kurtosis, and skewness of the measured variables. Since SEM assumes multivariate normality, analysis is conducted under the assumption that measured variables follow a normal distribution. If the absolute value of skewness in testing univariate normality is greater than 3.0, it is very likely ‘extremely ‘skewed. As for Kurtosis, if it is greater than 10.0, then the variable has failed the normality test. The kurtosis and skewness of the IADL among the measured variables were not normally distributed. The logarithmic transformation of IADL could be used to assume univariate normality with skewness of 2.61 and kurtosis of 7.67. However, the multivariate kurtosis was 15.43, which did not meet the assumption of multivariate normality; therefore, we used an ADF estimation that can effectively deal with the unassured data and a large sample size (≥ 500) [72].

**Table 2:** Descriptive statistics of the observed variables (N = 1022).

Variables	Mean±SD	Range	Skewness(log)	Kurtosis(log)
Hand grip strength	1.64±0.48	1–2	-0.59	-1.65
Timed up & go score	1.25±0.43	1–2	1.14	-0.70
Body mass index	1.76±0.43	1–2	-1.20	-0.56
K-IADL	7.74±1.73	7–22	3.62(2.61)	17.16(7.67)
Social activity participation (frequency)	1.87±1.28	1–4	0.55	-1.43
Number of neighbor contacts	3.39±1.31	1–5	1.97	2.21
Number of family contacts	2.90±1.39	1–5	1.04	-0.31
Economic activity participation (frequency)	0.76±1.26	`1–4	-1.12	-0.67
GDSSF-K	5.33±3.74	0–12	0.26	-1.18
RULS	36.86±12.73	18–72	0.58	-0.39
EQ-5D	0.74±0.18	-.17–1.0	-1.22	3.84
EQ-VAS	63.48±21.21	0–100	-0.43	-0.01
Multivariate				25.61(15.43)

EQ-5D: Euro QoL-5 Dimension-3 Level, EQ-VAS: EQ Visual Analogue Scale, GDSSF-K: Geriatric Depression Scale Short Form Korean Version, K-IADL: Korean Instrumental Activities of Daily Living, RULS: Revised UCLA Loneliness Scale

In this study, VIF, tolerance limits, and correlation coefficients were measured between independent variables to evaluate multicollinearity. The VIF was < 10 and tolerance limit > 0.1 among the independent variables, and the absolute value of the correlation coefficient between the measured variables was 0.00–0.62, which eliminated the problem of multicollinearity.

### Structural model

In the initial structural model testing, to solve the Haywood case of IADL and economic activity in which one measurement variable explains one potential variable, the error variance of the IADL was calculated as (1-α) X S2. The error variance of EAP was fixed at 0.005 [40]. After assessing the modification indices and parameter estimates, several paths in the hypothesized model were identified as being non-significant and deletion from and inclusion in the hypothetical model were carried out [40]. HGS and height were positively correlated [73], and HGS substantially matched height and weight [74] such that covariance between HGS (e1) and BMI (e3) was established. As there was a substantial relationship between the number of SAPs and neighbor contacts, we added the correlation of measurement error within the factor. The modified model revealed good overall fit (χ2/df [CMIN/df, Q] = 2.90, GFI = 1, AGFI = 1, RMSEA = .04, NFI = .86, IFI = .90, CFI = .90, PNFI = .51, and PCFI = .53). The fit criteria met the recommended level and were found to be more suitable than the hypothetical model (Table 3).

**Table 3:** Comparison of model fit of the modified model to the hypothetical model.

Type	Indices	Acceptable value	Fitness indices	
			Hypothetical model	Modified model
Absolute fit index	χ^2^		125.49	112.89
	*P*	> .1	0.000	0.000
	Q	≤ 3	3.06	2.90
	GFI	≥ .9	1	1
	AGFI	≥ .9	1	1
	RMSEA	≤ .08	0.05	0.04
Incremental fit index	NFI	≥ .9	0.84	0.86
	IFI	≥ .9	0.89	0.90
	CFI	≥ .9	0.89	0.90
Parsimonious fit index	PNFI	0–1	0.53	0.51
	PCFI	0–1	0.55	0.53

AGFI: Adjusted goodness of fit index, CFI: Comparative fit index, GFI: Goodness-of-fit index, IFI: Incremental fit index, NFI: Normed fit index, PCFI: Parsimony-adjusted comparative fit index, PNFI: Parsimony-adjusted normed fit index, RMSEA: Root mean square error of approximation.

**Fig 2:**
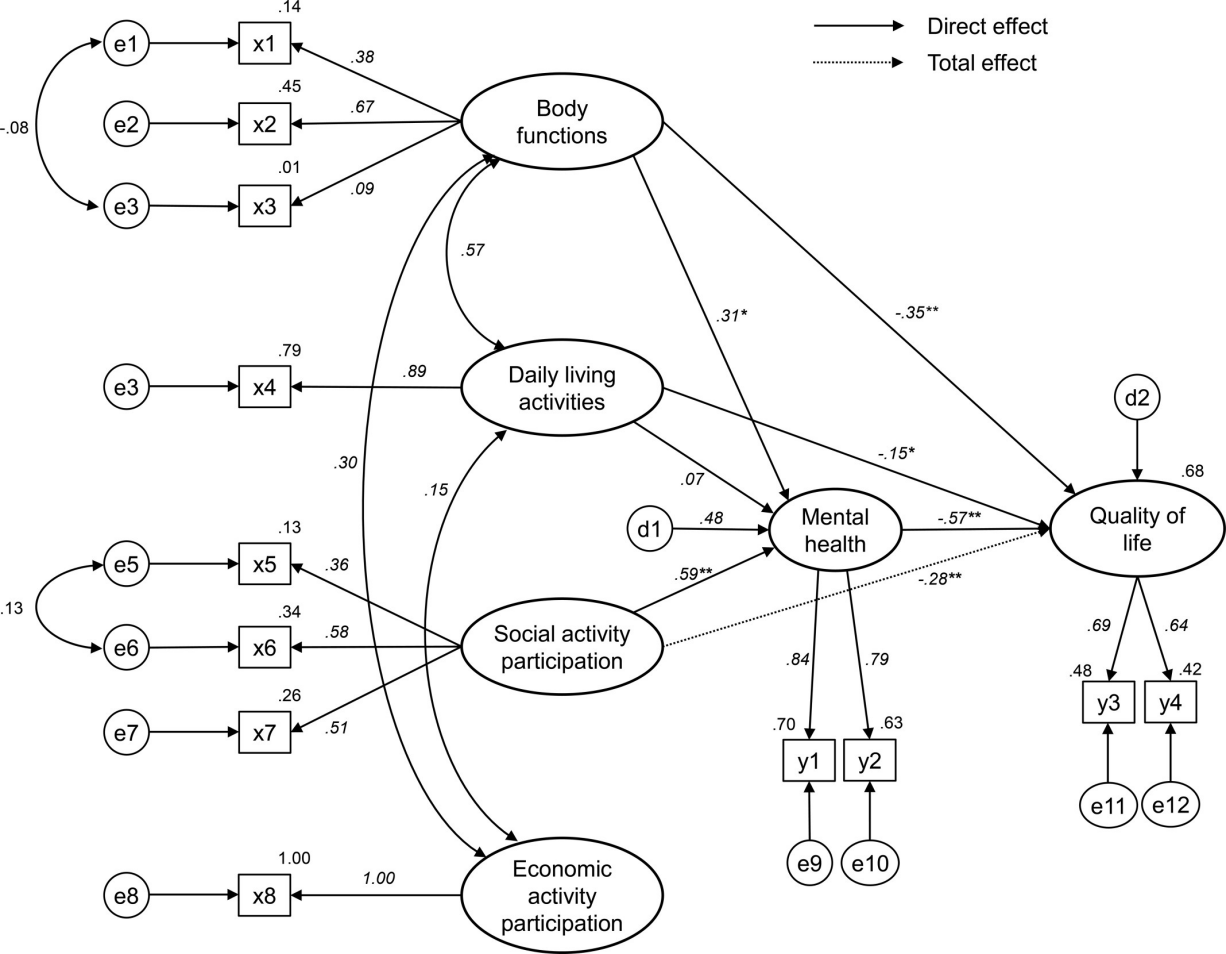
Path diagram of the modified model. *p < .05, ** *p* < .01, *** *p* < .001; x1 Hand grip strength; x2 Timed up and go score; x3 Body mass index; x4 Korean instrumental activities of daily living; x5 Social activity participation frequency; x6 Number of neighbor contacts; x7 Number of family contacts; x8 Economic activity participation frequency; y1 Geriatric Depression Scale Short Form-Korean; y2 Revised UCLA Loneliness Scale-version 3; y3 Euro Quality of Life-5 Dimension index; y4 Euro Quality of Life Visual Analogue Scale; e1-12 Measurement error in the Observed variables of x1-8 and y1-4; d1 Structural error in the Endogenous variable of Mental Health; d2 Structural error in the Endogenous variable of Quality of Life.

The final model demonstrated that body functions (β = .31, t = 3.78) and SAP (β = .59, t = 5.88) had a significant direct positive effect on mental health, with 48% being explained by the variables. Variables that significantly affected the QoL of older adults living alone were body functions (β = -.35, t = -3.74), daily living activities (β = -.15, t = -2.26), and mental health (β = -.57, t = 6.85), which together explained 68.3% of the variance in QoL. Standardized direct and indirect effects are reported in Table 4. Mental health had the greatest accumulated direct and total effect on QoL (β = -.57, p < .01). Body functions had the second greatest total effect on QoL (β = -.53, p < .01), both directly (β = -.35, p < .01) and indirectly (β = -.18, p < .01) through mental health. The direct effect (β = -15, p < .05) and total effect (β = -20, p < .05) of daily living activities on QoL was noted, but its indirect effect (β = -.04) through mental health was not statistically significant. SAP had a total effect (β = -.28, p < .01) and an indirect effect (β = -34, p < .01) on QoL through mental health. However, no effect of EAP was statistically significant. The structural model with standardized path estimates is presented in Fig 2.

**Table 4:** Standardized estimates of the modified model.

Endogenous variable	Exogenous variable	β	C.R. (t)^†^	*p*	SMC^‡^	Standardized direct effects	Standardized indirect effects	Standardized total effects
Mental	Body functions	0.31	3.78***	< .001	.48	0.31*	0	0.31*
health	Daily living activities	0.07	1.0	0.321		0.07	0	0.07
	Social activity participation	0.59	5.88***	< .001		0.59**	0	0.59**
	Economic activity participation	0.04	1.18	0.237		0.04	0	0.04
Quality of	Body functions	-0.35	-3.74***	< .001	.68	-0.35**	-0.18**	-0.53**
life	Daily living activities	-0.15	-2.26*	< .05		-0.15*	-0.04	-0.2*
	Social activity participation	0.06	0.63	0.530		0.06	-0.34**	-0.28**
	Economic activity participation	0.01	0.40	0.691		0.01	-0.03	-0.01
	Mental health	-0.57	-6.85***	< .001		-0.57**	0	-0.57**

*p < .05

** *p* < .01

*** *p* < .001 (†C.R.: Critical ratio

‡SMC: Squared multiple correlations).

## Discussion

This study tested a model with the aim of explaining and predicting the QoL of older adults living alone through determining how QoL is related to functional abilities and mental health. This modified model accounted for 68.2% of the variance in QoL. Factors that affected QoL were body functions, daily activities, SAP, and mental health.

Mental health exhibited the strongest direct impact on QoL; QoL declined as mental health deteriorated. Our results are consistent with previous findings on depression [75] and loneliness [37,76]. As individuals grow older, they become lonely and isolated in their homes (social isolation) and may be predisposed to more loneliness and depression [31]. Older adults living alone are less likely to share their thoughts and experiences and resolve depression and loneliness, which might be due to a lack of financial, physical, and emotional support from their family or friends [77]. Thus, depression and loneliness may be strong contributors to QoL. As older adults living alone may have dominant negative emotions and vulnerabilities, interventions to improve their QoL should include screening tests and strategies for mitigating depression and loneliness. Mental health could also have a negative impact on QoL as an intervening variable through reduction of body functions and SAP. SAP has a substantial impact on mental health. Previous studies have also shown the link between better mental health and frequent SAP [30,78]. However, the hypothesis that SAP has a direct effect on QoL was not supported in this study. This contradicts previous research examining the relationship between SAP and QoL [79], but another study [80] showed results similar to ours. Cho and Yoo [79] measured SAP using three aspects: type and level of, attitudes toward, and satisfaction with SAP. Lee [80], like us, measured SAP through only the frequency of contact with others, neighbors, family, and social activity. Levasseur, Richard, Gauvin, and Raymond [81] proposed the definition and classification of SAP according to the level of participation because the lack of clarity about the definition of SAP leads to communication obstacles between researchers, choice of research measurements, and research results comparison issues, as well as potentially inefficient social policies. More research is needed to clarify the mechanisms behind the link between SAP and QoL. Prior to this, a clear definition of SAP and the development of standardized measurement tools are required. This study emphasized the role of SAP as a predictor of mental health. As mental health problems that may be caused by living alone such as social isolation, reduced social relations, depression, lack of confidence, and loneliness [13,30] can be reduced by continuous SAP [76,78,79,82], mental health intervention programs that involve SAP should be developed.

Body functions were shown to have the second largest path to QoL and an indirect impact on QoL through mental health, an intervening variable, meaning that low objective levels of body functions (the muscle strength, flexibility, balance, cardiopulmonary endurance, etc.) can support the possibility of depression [83], and decreased mental health and, as a consequence, result in lower QoL [83,84]. However, there is limited research on objective body functions [83]. Objective body functions are physical abilities involving physique, posture, form and strength, agility, endurance, and equilibrium [83]. This study is substantially significant in that it confirmed the path of mental health and QoL through the measurement of objective body functions such as HGS, mobility and balance, and BMI. In older adults, the progression of deterioration in body function develops into a disability [85] and acts on changes in mental health such as lack of confidence, loneliness, and depression [13]. Body functions are an important predictor of QoL of older adults [8]. As maintenance and enhancement of body functions of older adults is closely related to their QoL, an evaluation system to check objective body functions of community-dwelling older adults and training of professional personnel to measure and evaluate them are needed. It is crucial, at both the individual and societal levels, to understand and manage the individual body functions through customized disease prevention and health promotion programs, as the number of older adults with good body function levels is increasing despite the rising prevalence of chronic diseases [38].

Daily activities did not have an indirect path through the intermediation of mental health; rather, they were found to directly affect QoL. A previous study that examined the depressive influencing factors of low-income older adults based on the ICF model [86] has also not shown a link between daily life activities/instrumental daily life activities and depression. The result was also consistent with research that found a positive correlation between IADL and QoL of older adults [87,88]. A two-year multidisciplinary longitudinal study of people aged 50 to 60 in Europe [89] showed that body functions are more important in predicting mental health than daily activities. However, another study [88] identified that daily activities among community-dwelling older adults affected depression and QoL. Park [88] reported that the ability to perform IADL has a negative correlation with depression and positive correlation with QoL. Participants who were completely independent of IADL were 68.5% in this study and 58.8% in Park’s study. Participants in our study reported that they performed better in IADL than participants in Park’s study [88]; therefore, it is thought that the effect mediating negative mental health such as depression and loneliness was not statistically significant. The ability to perform daily living activities is an essential element for independent living, and disability in daily living activities can advance the admission of older adults living alone in facilities owing to requiring the support of others; therefore, continuous evaluation and appropriate support should be provided. It is necessary to develop a strategy of community-centered specific management programs to ensure that professionals who can assess and mediate individual activity levels of the target audience are able to detect changes in the level of activity early among community-dwelling older adults and provide systematic support.

No statistically significant pathway was found between mental health and EAP or between EAP and QoL. This finding was consistent with a study that found that job participation for low-income older adults was not a factor affecting depression [86], but it contradicted other studies [58,59,82,90] that found EAP to improve mental health. The degree of economic activity participation of older adults in 28 OECD countries (United States 17.7%, Canada 12.9%, France 2.3%, Japan 20.8%, etc.) was lower than that in our study (27.4%) [91]. In a study of Americans over the age of 50, Manacy [58] reported that 34% of older adults received opportunities to meet social expectations as well as regular opportunities for exchange with others through EAP, which granted them social status, thereby improving their mental health. In a study of Japanese older adults over the age of 65, Tomioka et al. [90] reported that among women, the relationship between paid work and physical functioning and activity showed a positive relationship only for infrequent participation, 3 times or less per week. Kang and Kim’s [82] study showed that job participation has a significant impact on older adults’ mental health. For older adults who are forced to participate in economic activities due to economic needs, EAP can contribute to the deterioration of QoL; therefore, the characteristics of economic activity, such as the reason, type, and duration, are more important than whether or not to participate [92]. Therefore, this difference in results may be because our study analyzed only the frequency of EAP without identifying aspects such as type, duration, and satisfaction. Further research should consider EAP characteristics that may directly affect QoL.

The final model had an acceptable fit and was suitable for predicting the QoL of older adults living alone. This study will also be useful due to its use of a theoretical framework for comprehensive evaluation of older adults, as objective statistical measures were used, including measures for depression and loneliness, and the ICF model was used for functional ability. This study confirms the path of mental health and QoL through the measurement of objective body functions such as HGS, TUG score, and BMI. The results show that it is necessary to maintain and improve older adults’ functional ability through accurate measurement of body function. When planning nursing interventions to improve QoL, appropriate mental health promotion programs are important, including individual tailored activities that accurately identify the body functions and daily activities of individuals and encourage them to participate in adequate social activities.

### Limitations and future research

This study is a secondary data analysis study with the following limitations. First, the general characteristics, such as age, gender, and economic status of the participants, were not included in the hypothetical model. This was due to the study’s focus on functional abilities for the older adults and its intention of confirming the QoL of older adults living alone at various functional levels. The consideration of the principle of simplicity (Occam’s razor) and the focus on arbitral variations were also used to increase the value and descriptive power of the model [40]. However, it is necessary to consider the general characteristics that might affect the functions and activities of individuals. Second, this study did not include body structure, which is a main component of the ICF model on mental health and QoL. Body structure is the anatomical structure and form of the body, which requires imaging and was not measured from raw data. Older adults are prone to changes in body structure, from aspects such as joint deformation and chronic diseases, and the resulting physical and mental problems are significant. However, the measurement of body structure has limitations in community studies, such as economic aspects and unnecessary radiation exposure. In addition, most older adults regularly visit medical institutions and perform structural assessments due to one or more chronic diseases. Thus, efforts are needed to establish a more systematic community-oriented health-care management system that enables the sharing of clear and appropriate information through the connection between individual medical institutions and the community health management system used by older adults who are living alone. Finally, this original data was not specially designed to study QoL of older adults living alone, which limited the availability of variables of interest. This study only considered frequency when measuring SPA and EPA. Parts of the underlying concepts of “duration,” “type,” and “satisfaction of participation in the activity,” are lacking in the questionnaire we used to test SPA and EPA. While the Western welfare state pursued labor-linked welfare on the basis of various social insurance systems, including income security, Korea reflected elements such as “work-linked” and “productivity” in the design of the welfare system from the beginning without the basic welfare system being expanded. Unlike foreign countries, economic activities in old age are taken for granted in Korea. Therefore, interest in starting new economic activities after retirement and the causal relationship between life satisfaction tends to be emphasized more in Korea’s independent context. Many foreign studies have been conducted mainly on life satisfaction and job satisfaction during the transition to retirement, and many studies have been conducted on the impact of overall social participation (including social contribution activities) on life satisfaction since entering old age. However, there is a lack of a clear definition of social participation and appropriate measuring tools to accurately measure it. Therefore, further research is needed to develop and validate measurement tools based on the creation of clear definitions of SAP and EAP.

## Conclusions

Mental health, body functions, and daily living activities were the observed variables included in the final model, and they directly affected QoL. Body functions and SAP indirectly affected QoL through mental health. The final model points to the emphasis that should be placed on mental health, especially loneliness and depression, in caring for older adults living alone.

To improve the QoL of older adults who are living alone, measures to reduce depression and loneliness should include maintaining and promoting body functions, improving SAP, and encouraging daily activities. In particular, the various educational activities and interventions offered to support existing mental health should involve active screening for loneliness and depression. In addition, it is important to develop a community-based health-care system and active policy interventions to ensure the maintenance and promotion of basic functions among older adults. Social and policy interventions are needed to revitalize SAP. We suggest that follow-up studies be conducted on the development of emotional support programs, including promoting social participation and maintaining body functions.

## Supporting information

S1 Data (XLSX)
